# Causal associations between inflammatory bowel disease and primary biliary cholangitis: a two-sample bidirectional Mendelian randomization study

**DOI:** 10.1038/s41598-023-35785-2

**Published:** 2023-07-06

**Authors:** Jiaxi Zhao, Kaixin Li, Xiaoyang Liao, Qian Zhao

**Affiliations:** 1 Department of General Practice, General Practice Medical Center, West China Hospital, West China School of Medicine, Sichuan University, Chengdu, Sichuan China; 2 Huadong Hospital Affiliated to Fudan University, Shanghai, 200040 China

**Keywords:** Physiology, Psychology, Diseases, Endocrinology, Gastroenterology, Risk factors

## Abstract

Inflammatory bowel disease (IBD) has been reported to be associated with hepatobiliary diseases. Previous observational and Mendelian randomization (MR) studies have suggested a causal association between IBD and primary sclerosing cholangitis (PSC). However, it is unclear whether IBD has a causal association with primary biliary cholangitis (PBC): another autoimmune liver disease. We obtained genome-wide association study (GWAS) statistics from published GWASs for PBC, UC, and CD. We screened qualified instrumental variables (IVs) based on the three major assumptions of MR. To determine the causal relationships between UC or CD and PBC, two-sample MR analyses were performed using inverse variance-weighted (IVW), MR-Egger, and weighted median (WM) methods, and sensitivity analyses were conducted to validate the robustness of the results. We also conducted reverse MR analysis to reveal the causal association between PBC and UC or CD. UC was associated with a higher risk of PBC (OR 1.35, 95% CI 1.05–1.73, *P* = 0.02) in the IVW method, and CD was associated with an increased risk of PBC (OR 1.18, 95% CI 1.03–1.36, *P* = 0.02) in IVW. The weighted median and MR-Egger regression of both diseases showed a consistent direction but were not statistically significant. Results of the reverse MR analysis did not suggest genetic susceptibility that PBC was associated with an increased risk of UC (OR 1.05, 95% CI 0.95–1.17, *P* = 0.34) or CD (OR 1.1, 95% CI 0.99–1.20, *P* = 0.06). The present study revealed that IBD subtypes could increase the incidence of PBC, but in turn, PBC did not increase the incidence of IBD subtypes. Understanding that IBD and PBC constitute mutual risk factors can help with the clinical management of both diseases.

## Introduction

Primary biliary cholangitis (PBC), formerly known as primary biliary cirrhosis, is an autoimmune liver disease characterized clinically by chronic cholestasis and histologically by non-suppurative destructive cholangitis. Primary sclerosing cholangitis (PSC) and autoimmune hepatitis (AIH) and PBC are all autoimmune liver diseases^1^. ɤ-glutamyl-transferase (GGT) and alkaline phosphate (ALP) levels, which represent hepatic bile acid excretion, are crucial for the clinical management of PBC^2,3^. While Ursodeoxycholic acid (UDCA) is the first-line therapy, it is ineffective in up to 40% of PBC patients^4^. Previous studies have showed that PBC was linked to extrahepatic immune-mediated diseases, such as Hashimoto's thyroiditis and rheumatoid arthritis^5^.

Inflammatory bowel disease (IBD) is a chronic, an idiopathic inflammatory disease of the gastrointestinal tract that mainly includes two categories, including Crohn's disease (CD) and ulcerative colitis (UC). The etiology of IBD is related to genetic, autonomic immune, endocrine, and other factors^6,7^. With a prevalence of more than 0.3% in Western countries, IBD has become a global disease, emphasizing the importance of IBD prevention and management^8^. As a type of autoimmune liver disease, PSC is strongly linked to IBD^9^. However, the connection between IBD and PBC has yet to be determined. In recent years, there have been some case reports of concomitant PBC and IBD^10,11^. However, there is currently limited research on the potential causal relationship between IBD and PBC, highlighting the need for further studies to confirm this connection.

Traditional observational studies' ability to infer causality is hampered by potential confounding and reverse causality. Mendelian randomization (MR) analysis detects and quantifies causality using genetic variations as instrumental variables (IVs). There are three key hypotheses that must be met: the IVs: (1) has a strong correlation with exposure, (2) independent of confounding factors between exposure and outcome, (3) influences outcome directly through exposure^12,13^. This study conducted a two-sample bidirectional MR analysis using data from genome-wide association studies (GWASs) to assess the causal relationship between IBD subtypes and PBC.

## Materials and methods

### Study design

**Figure 1:**
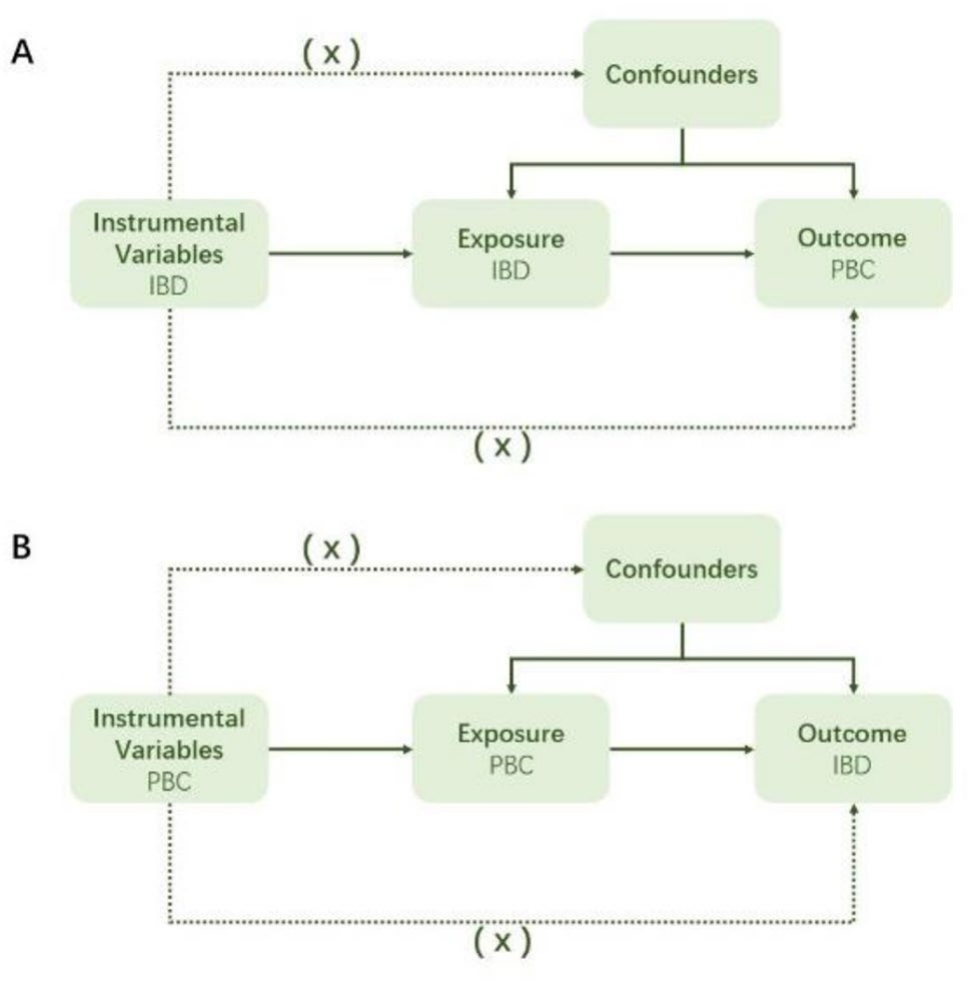
The flow diagram of the MR analysis. (**A**) Using IBD SNPs as IVs to study the causal impact of IBD subtypes on PBC. (**B**) Using PBC SNPs as IVs to study the causal inpact of PBC on IBD subtypes. Solid lines indicate IVs (SNPs) are associated with exposure and can only influence outcome through exposure. Dashed lines indicate IVs (SNPs) are independent of any confounding variables between exposure and outcome. The confounders includes exposure to environmental pollutants, radiation, infectious agents, chemical xenobiotics, smoking, and previous pregnancies.

The study design involved a bidirectional Mendelian randomization (MR) analysis to assess the causal relationship between IBD subtypes (CD and UC) and PBC. Three key hypotheses were tested: (1) IVs were highly correlated with IBD subtypes, (2) IVs were independent of confounding factors between IBD subtypes and PBC, and (3) IVs did not influence PBC through factors other than IBD subtypes. The first analysis investigated the causal relationship between IBD subtypes as exposures and PBC as an outcome, and the second analysis investigated the reverse causality, using PBC as an exposure and IBD subtypes as outcomes (Fig. 1).

### Data source

The study utilized genetic association data from the International Inflammatory Bowel Disease Genetics Consortium (IIBDGC) GWAS. which included 42,950 cases with IBD and 53,536 controls without IBD. Specifically, data from 47,745 participants with UC (13,768 cases and 33,977 controls) and 51,874 participants with CD (17,897 cases and 33,977 controls) of European descent were included^14^. The UC and CD cohort populations were of European descent. Diagnosis of IBD and its subtypes was based on endoscopic, radiological, and histopathological criteria. Meanwhile, data on PBC were obtained from a meta-analysis of 2764 cases and 10,475 controls of European descent, diagnosed using the International Classification of Diseases (ICD-10) criteria.

The GWAS data used in this study were obtained from the IEU OpenGWAS online database, which provided access to a wide range of genetic data (accession number: ieu-a-970 for UC, ieu-a-12 for CD, and ebi-a-GCST003129 for PBC). The ethics committees of each of the participating centres or nations approved the use of data for UC and CD cohorts and all participants provided written, informed consent^14^.

### SNP selection

In this study, we identified single-nucleotide polymorphisms (SNPs) that were strongly associated with UC, CD, and PBC, with genome-wide significance of *P* $<$ 5 × 10^–8^. To ensure the independence of the SNPs, we eliminated those with linkage disequilibrium (LD) greater than r2 < 0.001 within a clumping distance of 10,000 kb. We then assessed the strength of the instrumental variables (IVs) using an F statistic > 10 to minimize the impact of weak IVs on the causal analysis. The F statistic was calculated using the following equation: F = (R^2^/1 − R^2^)(n − k − 1/k), where n is the sample size and R^2^ is the variance as interpreted by the IVs. R^2^ was calculated using the minor allele frequency (MAF) and β-value: R^2^ = 2MAF (1 − MAF) β^2^. All variables used in the analysis had F values greater than 10. By harmonizing, we also eliminated ambiguous and palindromic SNPs. To avoid potential pleiotropy, we used the PhenoScanner V2 (http://www.phenoscanner.medschl.cam.ac.uk/) to exclude IVs associated with confounding or risk factors for PBC, such as exposure to environmental pollutants and radiation^15^. In total, 43, 59, and 16 SNPs were used as IVs for UC, CD, and PBC for subsequent MR analysis. Details of these IVs are provided in Supplementary Tables 1–3, and all the confounders and excluded SNPs are in Supplementary Table 7.

### Mendelian randomization analyses

We combined several statistical methods in the MR analysis. The primary method was the inverse variance weighted (IVW), which is with balanced pleiotropy that is expected to be stable. In addition, MR-Egger regression, weighted median, and MR pleiotropy residual sum and outlier (MR-PRESSO) test were used as supplements to the IVW method to estimate the causal relationship under different conditions^16–18^. The results were reported as odds ratios (ORs) and 95% confidence intervals (CIs). and statistical significance was considered at *P*-value < 0.05. All statistical analyses have been performed using the TwoSampleMR package (version 0.5.6) in R (2022.02.3)^19^.

### Sensitivity analysis

Horizontal pleiotropy occurs when IVs associated with the exposure influence the outcome through multiple factors other than the exposure. To assess the presence of horizontal pleiotropy, we used the MR-Egger intercept test, where a significant intercept (*P* < 0.05) indicates the presence of pleiotropy, and the results should be interpreted with caution. The results of MR-Egger intercept test were visualized by scatter plots. We also used Cochran's Q statistics to examine heterogeneity, where significant heterogeneity (*P* < 0.05) indicates the presence of heterogeneity among the included studies, and the results were visualized using funnel plots. In order to remove horizontal pleiotropy, we use MR-PRESSO outlier test to remove abnormal SNPs (outliers) and estimate the corrected results. In the leave-one-out analysis, results were re-analyzed after removing SNPs once at a time, and forest plots were drawn to intuitively judge the rubustness of the results.

## Results

### Mendelian randomization analyses

#### Effect of UC or CD on PBC

According to the IVW method, UC was associated with a higher risk of PBC (OR 1.35, 95% CI 1.05–1.73, *P* = 0.02). Both MR-Egger regression and weighted median showed a consistent direction but insignificanct results (OR 1.12, 95% CI 0.43–1.95, *P* = 0.824 and OR 1.17, 95% CI 0.99–1.38, *P* = 0.057, respectively).

In the IVW method, CD was associated with an increased risk of PBC (OR 1.18, 95% CI 1.03–1.36, *P* = 0.02). The weighted median (OR 1.13, 95% CI 0.99–1.28,* P* = 0.056) and MR-Egger regression (OR 1.17, 95% CI 0.81–1.69, *P* = 0.413) showed a consistent direction but not statistically significant results (Supplementary Table 4).

#### Effect of PBC on UC or CD

We also conducted a reverse MR analysis between PBC and IBD subtypes, but the IVW method did not reveal any reverse causal relationships. Supplementary Table 5 provides the results of the reverse MR analysis.

### Sensitivity analysis

We conducted Cochran's Q statistics and its funnel plots, the MR-Egger intercept test and its scatter plots, the leave-one-out analysis and its forest plots in the sensitivity analysis. Moreover, MR-PRESSO test was used to correct for heterogeneity. The analyses of UC on PBC revealed significant heterogeneity (Q = 270.452, *P* = 9.660 × 10^–35^ for IVW). There was no evidence of directional pleiotropy (intercept = 0.0422, *P* = 0.295). (Supplementary Table 6) After outliers were removed, the association was significant (outlier-corrected: β = 0.14, 95% CI 0.014–0.295, *P* = 0.031), and the value of NbDistribution was 10,000. The leave-one-out analyses demonstrated the robustness of the findings.

There was significant heterogeneity in Cochran's Q statistics between CD and PBC (Q = 192.294, *P* = 1.445 × 10^–16^). But there was no evidence of pleiotropy between CD and PBC (intercept = 0.0013, *P* = 0.956). (Supplementary Table 5) To correct the heterogeneity, we removed the outliers and conducted the MR-PRESSO test, the results showed that the association was significant (outlier-corrected: β = 0.14, 95% CI 0.028–0.252, *P* = 0.015), and the value of NbDistribution was 10,000.

**Figure 2:**
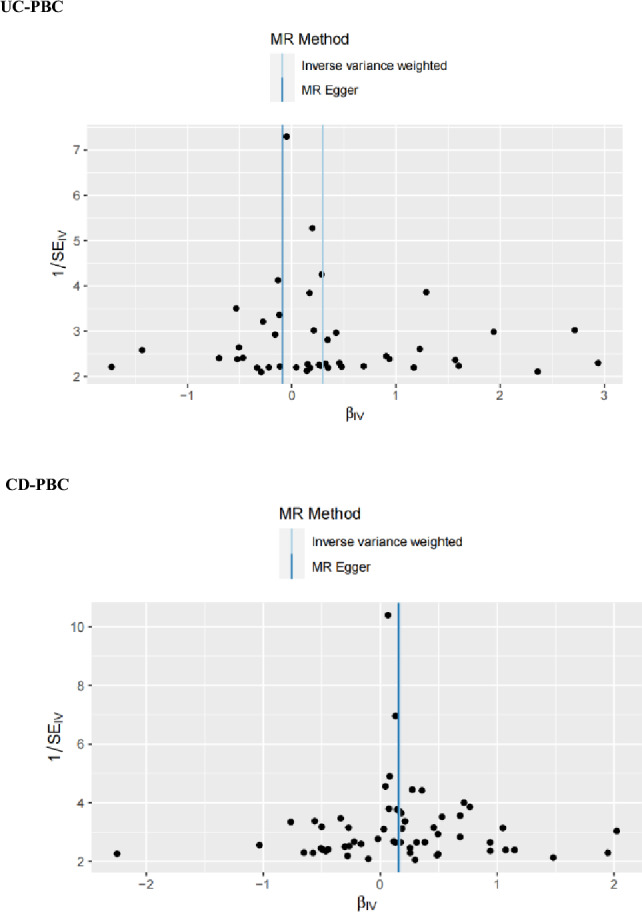
Causal relationships between UC or CD and PBC in funnel plots. *UC* ulcerative colitis, *CD* Crohn’s disease, *PBC* primary biliary cholangitis, *MR* Mendelian randomization.

**Figure 3:**
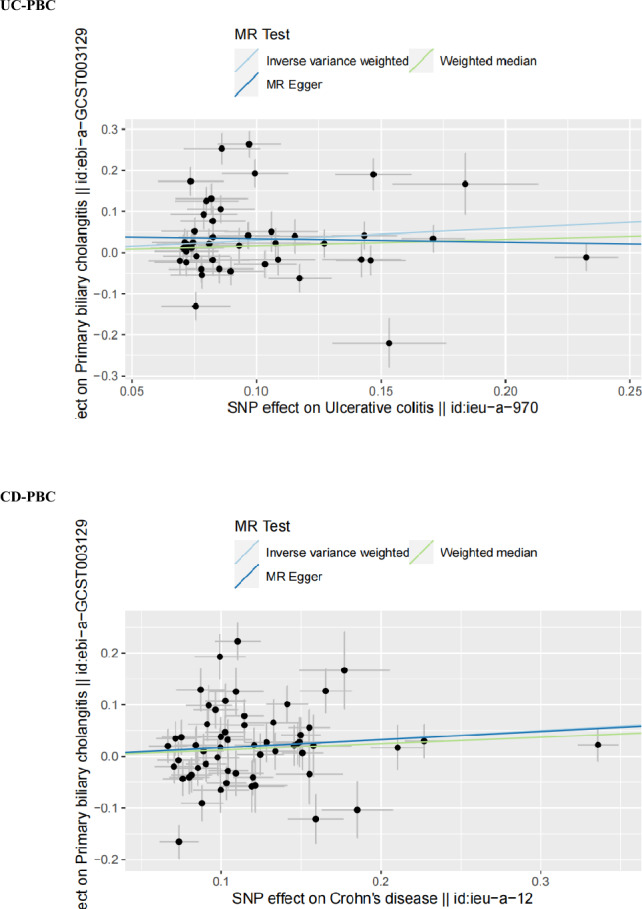
Causal relationships between UC or CD and PBC in scatter plots. *UC* ulcerative colitis, *CD* Crohn’s disease, *PBC* primary biliary cholangitis, *SNPs* single-nucleotide polymorphisms, *MR* Mendelian randomization.

**Figure 4:**
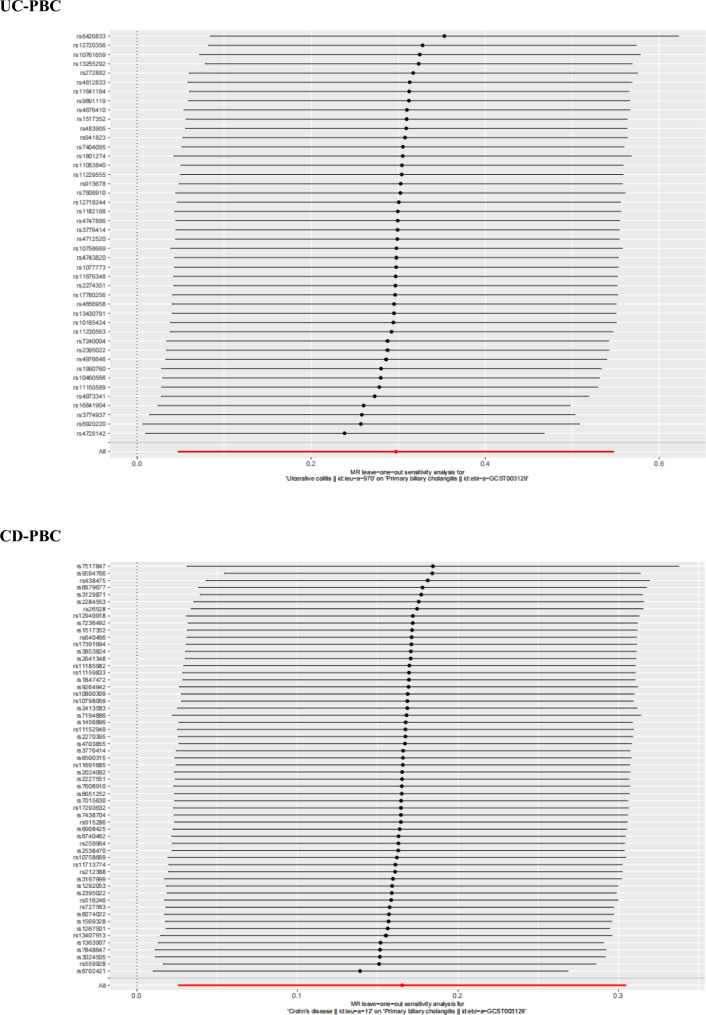
Forest plots of SNPs associated with UC or CD and risk of PBC. *UC* ulcerative colitis, *CD* Crohn’s disease, *PBC* primary biliary cholangitis, *SNPs* single-nucleotide polymorphisms, *MR* Mendelian randomization.

The funnel plots of the MR analysis' Cochran's Q statistics are displayed in Figs. 2, 3 and 4 depicted the scatter plots of the causal relationships between IBD subtypes and risk of PBC, as well as the forest plots of the leave-one-out analyses, respectively.

The IVW method in the MR analysis revealed no reverse causality between PBC and UC or CD. As a result, additional heterogeneity and pleiotropy testing was not required.

### Permission to reproduce material from other sources

The authors of the GWAS data have approved the use and citation of the two sets of GWAS data included in the article.

## Discussion

The etiology of PBC, an autoimmune liver disease, remains unclear, and treatment response is often unreliable. Therefore, it is crucial to investigate diseases that may increase the risk of developing PBC. Building on the established causal relationship between IBD and PSC, this study explores the potential causal relationship between IBD and PBC, offering novel insights into the prevention and treatment of PBC. By identifying the causal association between these two diseases, this study provides a starting point for future investigations into the shared underlying mechanisms and potential therapeutic targets. Ultimately, this work may help improve patient outcomes and inform clinical decision-making in the management of PBC.

In this study, we utilized GWAS data from the IIBDGC large-scale and a PBC meta-analysis to investigate the possible causal relationship between UC or CD and PBC susceptibility. Multiple MR methods were employed, and reverse MR to investigate the relationship between PBC and UC or CD susceptibility. The primary MR analysis indicated that both UC and CD were causally associated with an increased risk of PBC (OR 1.35, 95% CI 1.05–1.73, *P* = 0.02; OR 1.18, 95% CI 1.03–1.36, *P* = 0.02, respectively). However, reverse MR analysis did not find a causal relationship between PBC and IBD subtypes.

Both UC and CD were linked to a variety of hepatobiliary symptoms^20^. PSC is the best known, and nonalcoholic fatty liver disease (NAFLD) is the most common. A study published in 1999 by Koulentaki M found that the prevalence of PBC in IBD patients was higher than in the general population^21^. In a clinical study of six patients with IBD and PBC, PBC was diagnosed after. (the mean time between IBD and PBC diagnosis was 7.1 years, ranging from 1.1 to 22.2 years)^11^.

**Figure 5:**
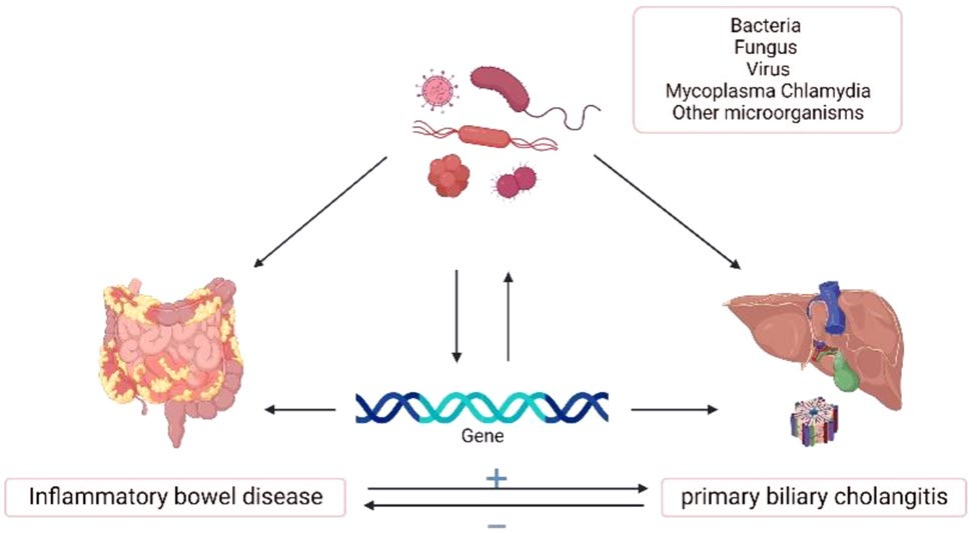
The relationships between infection, genetic code, IBD and PBC. Infection can directly affect the occurrence of IBD and PBC, and can also lead to susceptibility to IBD and PBC by affecting genetic changes. Genes also cause susceptibility to certain infections; IBD may increase the incidence of PBC, but PBC did not increase the incidence of IBD. *IBD* Inflammatory bowel disease, *PBC* primary biliary cholangitis.

Genetics is a crucial factor in the association between IBD and PBC. The association of UC and PBC with genes on the short arm of chromosome 6 may imply that the inflammation genes play a pathogenic role in both diseases. Certain gene variants linked to PBC may predispose individuals to specific infections, and the studying the genetic components of PBC could shed light on associations with infectious agents. Surprisingly, recent epidemiological studies have repeatedly shown a link between PBC and infectious agents^22–24^. More than 200 risk gene loci are known to be associated with IBD, including genes related to susceptibility to infection^14,25^. The shared presence of major histocompatibility complex (MHC) genes in both diseases also highlights their potential genetic overlap^26,27^. In terms of immunology, PBC's autoimmune nature is supported by increased immunoglobulin levels, activated T lymphocytes, and immune complex formation, while IBD's autoimmune nature is revealed by autoantibodies against intestinal epithelial cells and leukocytes, as well as its association with human leukocyte antigen (HLA) haplotypes and response to corticosteroid therapy^28,29^ (Fig. 5).

Despite the potential link between IBD and PBC, the current clinical studies on their relationship remain limited, the available studies mainly consist of case reports, including one in which a patient developed PBC after having a colectomy, implying that surgery may be a factor in promoting disease progression^30^. Gut permeability disruption in IBD may also lead to bacterial translocation, cholangiocyte activation, and liver inflammation, ultimately leading to the development of PBC^31^. When hepatic insufficiency occurs in IBD patients, it is important to determine whether PBC is present. Improvements in serum anti-mitochondrial antibody (AMA), alkaline phosphatase (ALP), and liver histopathology can help confirm the diagnosis of PBC^32,33^. To gain a better understanding of the causal association between IBD and PBC, long-term prospective studies are necessary. As research progresses, further clarification of the mechanism behind the association between these two diseases can aid in clinical prevention.

We want to highlight some of our study's strengths while also acknowledging some of its limitations. On the one hand, this was the first study to use the 2-sample MR method to assess bidirectional causality between IBD and PBC. This approach had the advantage of being less susceptible to confounding factors, and reverse causality when compared to observational studies and intervention experiments. On the other hand, we studied the rare clinical disease of IBD combined with PBC in a novel way, going beyond the limitations of previous case reports. The study also had limitations. First, due to data availability, we limited the population to people of European ancestry. As a result, applying the findings of this study to other populations should be careful. Second, while removing linkage disequilibrium and detecting pleiotropy were used in the selection and processing of IVs to minimize and monitor their effects on the results, it took much work to avoid them altogether. Furthermore, the MR analysis was based on summary-level data, and individual-level data were not available. As a result, the possibility of residual confounding or other limitations inherent in such data cannot be entirely ruled out. Finally, while MR analysis can suggest a potential causal relationship, it cannot establish causality definitively. Therefore, the results of this study should be interpreted with caution and further validated through additional studies, including experimental studies. Despite these limitations, our study provides new insights into the relationship between IBD and PBC and offers potential avenues for further investigation and future research.

## Conclusions

This study represents the first attempt to use MR analysis to explore the bidirectional causal relationship between IBD and PBC. Our findings suggest that IBD may increase the incidence of PBC, but not vice versa. These results underscore the need for further investigation into the underlying mechanisms linking IBD to the development of PBC.

## Supplementary Information

Supplementary Information.

## Data Availability

The datasets used or analyzed during the current study are available from the corresponding author upon reasonable request.
